# The mental health care system for children and adolescents in Greece: a review and structure assessment

**DOI:** 10.3389/frhs.2024.1470053

**Published:** 2024-12-11

**Authors:** Lauro Estivalete Marchionatti, Julia Luiza Schafer, Vasiliki Eirini Karagiorga, Panagiota Balikou, Andromachi Mitropoulou, Aspasia Serdari, Giorgos Moschos, Lilian Athanasopoulou, Maria Basta, André Simioni, Julian Vicenzi, Efstathia Kapsimalli, Alexandra Tzotzi, Sotiria Mitroulaki, Katerina Papanikolaou, Kalliopi Triantafyllou, Dimitra Moustaka, Shekhar Saxena, Sara Evans-Lacko, Christos Androutsos, Anastasia Koumoula, Giovanni Abrahão Salum, Konstantinos Kotsis

**Affiliations:** ^1^ Child and Adolescent Mental Health Initiative (CAMHI), Stavros Niarchos Foundation & Child Mind Institute, Athens, Greece; ^2^ Global Programs, Child Mind Institute, New York, NY, United States; ^3^ Department of Psychiatry, Universidade Federal do Rio Grande do Sul (UFRGS), Porto Alegre, Brazil; ^4^ Department of Child and Adolescent Psychiatry, Medical School, Democritus University of Thrace, Alexandroupolis, Greece; ^5^ Department of Psychiatry, University Hospital of Heraklion, Crete, Greece; ^6^ Department of Child and Adolescent Psychiatry, University Hospital of Heraklion, Crete, Greece; ^7^ Department of Child Psychiatry, Agia Sophia Children’s Hospital, National and Kapodistrian University of Athens, Athens, Greece; ^8^ Department of Psychology, Neapolis University Pafos, Paphos, Cyprus; ^9^ Department of Global Health and Population, Harvard T H Chan School of Public Health, Boston, MA, United States; ^10^ Care Policy and Evaluation Centre, London School of Economics and Political Science, London, United Kingdom; ^11^ Department of Child and Adolescent Psychiatry, Sismanoglio General Hospital of Attica, Athens, Greece; ^12^ Department of Psychiatry, Faculty of Medicine, School of Health Sciences, University of Ioannina, Ioannina, Greece

**Keywords:** mental health, public health, health system, child, adolescent, Greece

## Abstract

**Background:**

The mental health system in Greece faces challenges to complete its transition to a community-oriented model, having significant concerns for child and adolescent care due to lower coverage and service gaps. This component of the mental health system has not been comprehensively evaluated.

**Methods:**

We conducted a review of the mental health care system for children and adolescents in Greece. For a field assessment, we directly collected data from mental health services to map availability and distribution. We analyzed the needs of human resources using professional register data and the national census.

**Results:**

The National Health Care Service (ESY, *Εθνικ*ό *Σ*ύ*στημα Υγε*ί*α*ς) is the public health system in Greece, characterized by public governance but significant private participation. Although ESY aims for universal care, gaps in population coverage and high user fees create barriers to access. Embedded within ESY, the mental health system is shifting towards a community-oriented structure since the psychiatric reform. For children and adolescents, there is a developing framework for regionalization and community services, including day centers, inpatient facilities, outpatient departments, and school-based psychoeducational facilities. However, services lack coordination in a stepped care model. Patient pathways are not established and primary care rarely involves child mental health, leading to direct access to specialists. Services operate in isolation due to the absence of online registers. There is no systematic performance monitoring, yet some assessments indicate that professional practices may lack evidence-based guidelines. Our mapping highlighted a scarcity of public structures, with an unbalanced regional distribution and many underserved areas. Child and adolescent psychiatrists are predominantly affiliated with the private sector, leading to professional gaps in the public system.

**Conclusions:**

Our assessment identifies an established framework for a community-oriented, universally accessible mental health system, yet several barriers impede its full realization. These include an inconsistent primary healthcare system, a shortage of specialists in the public sector, imbalanced service distribution, lack of coordination among providers, underfunding, and absence of quality monitoring. We propose interventions to promote child and adolescent mental health in primary care, coordinate patient pathways, establish standards of care, and monitor performance.

## 1 Background

Mental and behavioral disorders have emerged as leading contributors to years lived with disability (YLDs) on a global scale, posing special concerns due to possible long-lasting impact on children and adolescents (1, 2). In Greece, the impact of mental health is further compounded by socioeconomic backdrops and limited capacity of the healthcare system (3, 4). There are approximately 1.4 million people in the country struggling with mental health conditions, accounting for about 12% of population (5). Mental health disorders are among top-ranked conditions that impact children's and adolescents' lives in Greece, constituting a substantial 24.6% of disability-adjusted life years lost to all diseases (DALYs) within the 5–14 age group. It is estimated that up to 11% of adolescents present mental health symptoms beyond the cut-off for professional evaluation, and the Covid-19 pandemic may have further exacerbated these figures (6–9).

The capacity of the healthcare system was strained by the financial crisis affecting the country over the last decade. From 2009 to 2015, cuts in public health expenditure reached 6.7 billion euros, mainly related to a reduction in Social Health Insurance (SHI) (10). Consequently, access to healthcare services deteriorated, and a substantial portion of the population lost coverage due to unemployment and to the increased load of out-of-pocket (OOP) payments (11, 12). Socioeconomic issues also take a toll on the mental health of people living in Greece: among adolescents, low family income was associated with depression and suicidality, and food insecurity elevated emotional symptoms (13–15). This underscores specific concerns for vulnerable groups, such as refugees living in harsh conditions with limited access to health care and at greater risk of mental health issues, with potential impacts on their psychosocial development and future prospects (16–18).

In light of this scenario, we conducted a narrative review of the mental healthcare system providing assistance to children and adolescents in Greece and an assessment of available structure through a nationwide mapping of services providing care to children and adolescents. In response to caveats identified in the system, we review possible strategies that have proven effective in similar contexts. This work is part of the Child and Adolescent Mental Health Initiative (CAMHI), a partnership between the Stavros Niarchos Foundation (SNF), the Child Mind Institute (CMI) and multiple institutions in the public sector in Greece to enhance the health care capacity for this particular population within the country.

## 2 Materials and methods

This work employs a narrative review and a nationwide mapping of available structure at service level to assess the current state of the child and adolescent mental health system in Greece. The review is based on a similar effort performed in Brazilian public mental health provision (19). We begin by examining the overarching National Health Care Service (ESY), including its governance, financing, and organizational structure. We then delve into the mental health care system embedded within ESY, detailing its structure, organization, available services, human resources, and coverage, with a particular focus on child and adolescent care.

We included academic articles and a comprehensive array of non-academic sources with relevant information on one or more aspects of child and adolescent mental health provision, including governmental and institutional reports, law and regulation, official census statistics, and records from professional associations and registers. An initial set of studies was identified during our previously reported systematic review of child and adolescent mental health in Greece, conducted through searches in PubMed, Web of Science, PsycINFO, Google Scholar, and IATPOTEK from inception to December 16th, 2021 (20). As a supplementary component of that work, we collected potentially relevant articles on the mental health system. While this set was not systematically reviewed, it provided a foundational reference for the present narrative review, facilitating further identification of pertinent literature and emerging topics. This process was further enriched by targeted Google Scholar and PubMed searches, as well as non-academic publications with valuable data (e.g., number of professionals registered in medical associations). Additionally, national protocols, guidelines, institutional reports, and legislative documents were compiled with input from local experts. In the recommendations section, we specifically reviewed scholarly articles from the international literature to identify effective strategies potentially pertinent to the Greek context.

For the nationwide mapping of services providing care to children and adolescents, we mapped public and philanthropic mental health and psychoeducational services for children and adolescents in each health sector between December, 2021 to May, 2022 with continuous revision throughout 2023 and 2024. We initially collected data at governmental and regional websites and then directly reached out to facilities for gathering information on service's type, location, operational status, and contact information (in the case of special schools, we have only consulted official lists). Next, experts from each health region revised the table and have been continuously checking updates. For generating population rates, we matched our collected data with population census. Data generated at this work is openly available in Open Science Framework at http://doi.org/10.17605/OSF.IO/CRZ6H.

## 3 Health system

**Figure 1:**
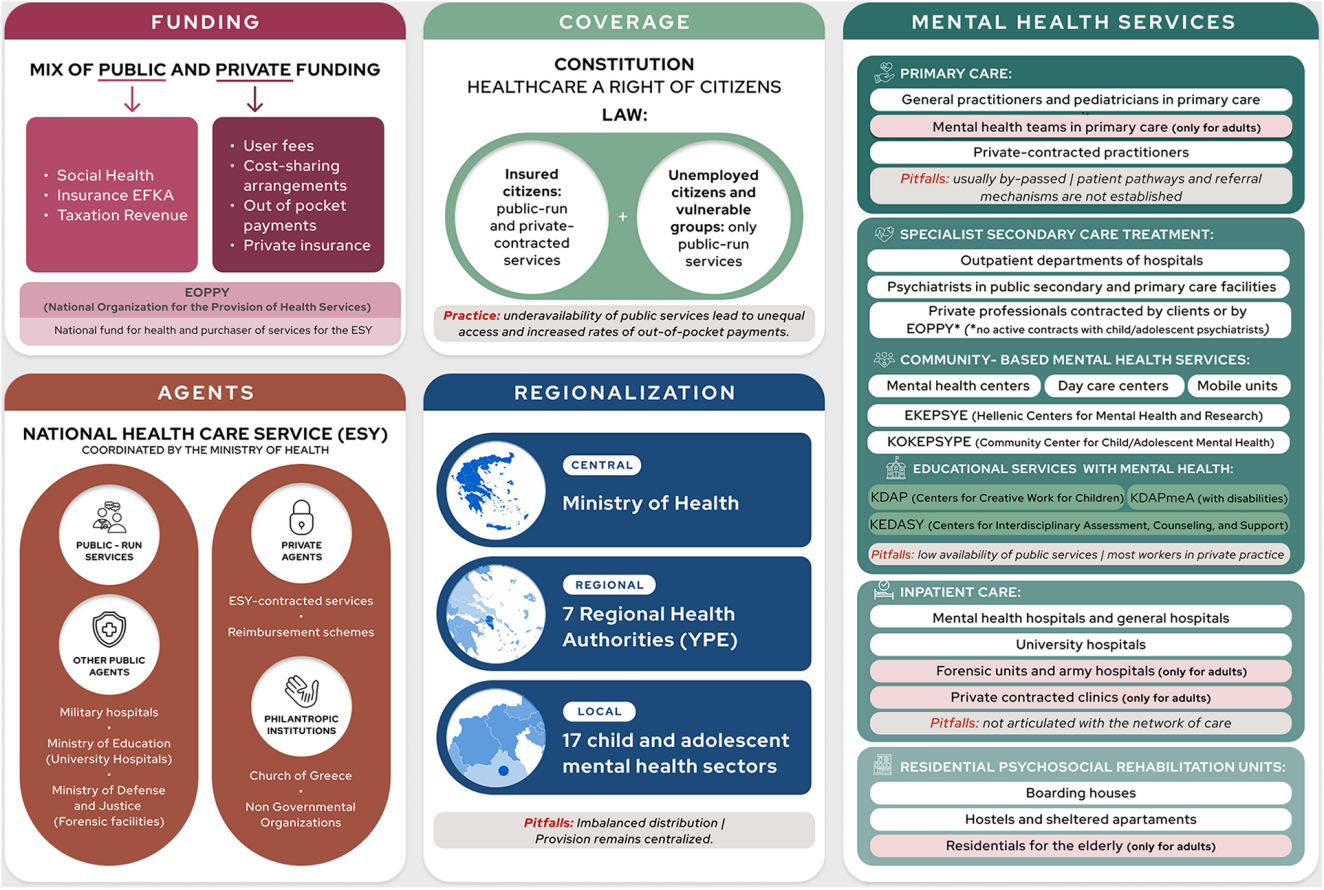
The health system framework and available services for mental health focusing on children/adolescents.

In the Hellenic Republic, healthcare is a constitutional right for citizens and a duty of the state (21). Services are delivered through a mix of public and private provisions and funding (see Figure 1 for a general framework). In the early 80s, the public health system was established as the National Health System (ESY, *Εθνικ*ό *Σ*ύ*στη*μ*α Υγε*ί*α*ς), functioning under the responsibility of the Ministry of Health and providing assistance across all levels of care (10, 22). The ESY operates its own facilities and workforce, also contracting private providers to supply services within the public framework. The private sector also operates independently through health insurance or direct payments, although under the regulation of public governance.

To a lesser degree, other stakeholders outside the ESY participate in the provision of health services in Greece (10). A few military hospitals are run by the Ministry of National Defence, occasionally extending services to the civilian population. Two teaching hospitals in Athens are operated by the Ministry of Education, Religious Affairs and Sport. The Church of Greece and Non-Governmental Organizations (NGOs) operate philanthropic facilities such as social medical centers, pharmacies, nursing homes, orphanages, community health clinics, and mobile units addressing the needs of vulnerable populations (23).

### 3.1 Coverage

Although the Constitution grants citizens the right to free and equitable health care, there are gaps of coverage (21, 24). Historically, health coverage in Greece has been linked to employment status through mandatory social insurance (guaranteeing access to all services provided within the public system) and to Greek/European Union citizenship (permitting access to some public services including primary and outpatient care) (10, 25, 26). This way, a disparity of coverage existed for vulnerable groups, such as uninsured citizens and refugees (27). In 2016, this was partially addressed by Law 4368, which extended access to ESY-operated services to those groups through an unemployment reserve, even though public services contracted from private entities remained excluded from this provision (28). On the practical level, there remain obstacles hindering access to health services for subgroups of the population (22, 26). Primarily stemming from inadequate funding for the ESY, they include insufficient availability of public services, a significant load of out-of-pocket payments for services contracted from the private sector, and suboptimal reimbursement arrangements (29–31). According to an European Observatory on Health Systems and Policies (OECD) (32) report, 56% of people in Greece were unsatisfied with coverage of health care services and 57,3% reported unmet long-term care needs.

### 3.2 Financing

The National Health System (ESY) is financed with a mix of public and private resources, and a considerable parcel is sourced from user fees (22, 33). Public financing originates from contributions to the Social Health Insurance (SHI) and taxation revenues (10, 34). The EFKA (*Ενια*ί*ο*ς *Φορ*έ*α*ς *Κοινω*ν*ικ*ής *Ασφ*ά*λιση*ς, meaning Single Social Security Fund) collects insurance contributions from employees and employers (including the state), allocating 7.1% of its income to EOPYY (the National Organization for the Provision of Health Services). The EOPYY acts as the exclusive purchaser of services in ESY and was established to unify previous healthcare insurance funds (35). The Ministry of Finance manages the funds, covering EOPYY deficits with the state budget. Over the years, Greece has consistently allocated a lower proportion of public funding to healthcare compared to the EU average, particularly in the aftermath of the financial crisis. In 2021, Greece's per capita healthcare expenditure was less than half of the EU average for that year (36).

Private expenditure is a major financing source, corresponding to up to 41% of investments in health (10, 29, 30, 32). It encompasses user fees for public services provided by private operators, cost-sharing arrangements for services and medications, out-of-pocket payments (OOP) for services outside the public system, and private insurance. There are reimbursable schemes for private services contracted by EOPYY, meaning users pay upfront and are eligible for a partial or total refund. There are some reports of hidden payments for privileged access to public services, which may amount to a considerable quarter of all private expenditure (30, 37).

### 3.3 Available services

Health services covered by the EOPYY are defined by The Integrated Health Care Regulation (EKPY), which specifies coverage, duration, and user charges for public-run and private-contracted services (10). It includes inpatient care, outpatient care, diagnostic procedures, therapeutic procedures (physical therapy, psychotherapy, speech therapy, occupational therapy, special education therapy), pharmaceutical care, dental care, obstetric care, and childbirth allowance. Table 1 summarizes user costs for services provided within ESY.

**Table 1:** Available health services and user costs.

Type of care	Provision	User costs
Outpatient care Consultations at primary and specialized care Laboratorial examinations Screening tests	Public facilities Outpatients departments of clinics and hospitals Private-contracted general practitioners and specialists Afternoon outpatient specialist visits to private patients in public hospitals^a^	Free of charge for facilities operated by the EOPYY Services contracted by EOPYY are free of charge but face a limit of 200 consultations per month and 20 consultations per day Services at private providers may be eligible for a partial reimbursement Afternoon outpatient specialist visits in public services present a user fee (€24,00 to €72,00)
Inpatient care	Public hospital, including university hospitals Private clinics	Free at public hospitals (may include user charges for extended services) Private clinics include a standard 30% user charge
Diagnostic and laboratory tests	Public and private providers	Free at public hospitals Co-insurance of up to 15% in contracted providers
Outpatient medications		Cost-sharing arrangements from 0% (for specific groups) to 25% (usual charge)
Mobile emergency care (prehospital)	Mobile units of the National Centre for Emergency Care in Greece (EKAV)	Free of charge

^a^ Physicians working for public hospitals can perform private consultations in the afternoons in the hospital. University and military are allowed private practice. Since 2024, ESY physicians can also have private practice (38).

### 3.4 Organization and patient pathways

**Figure 2:**
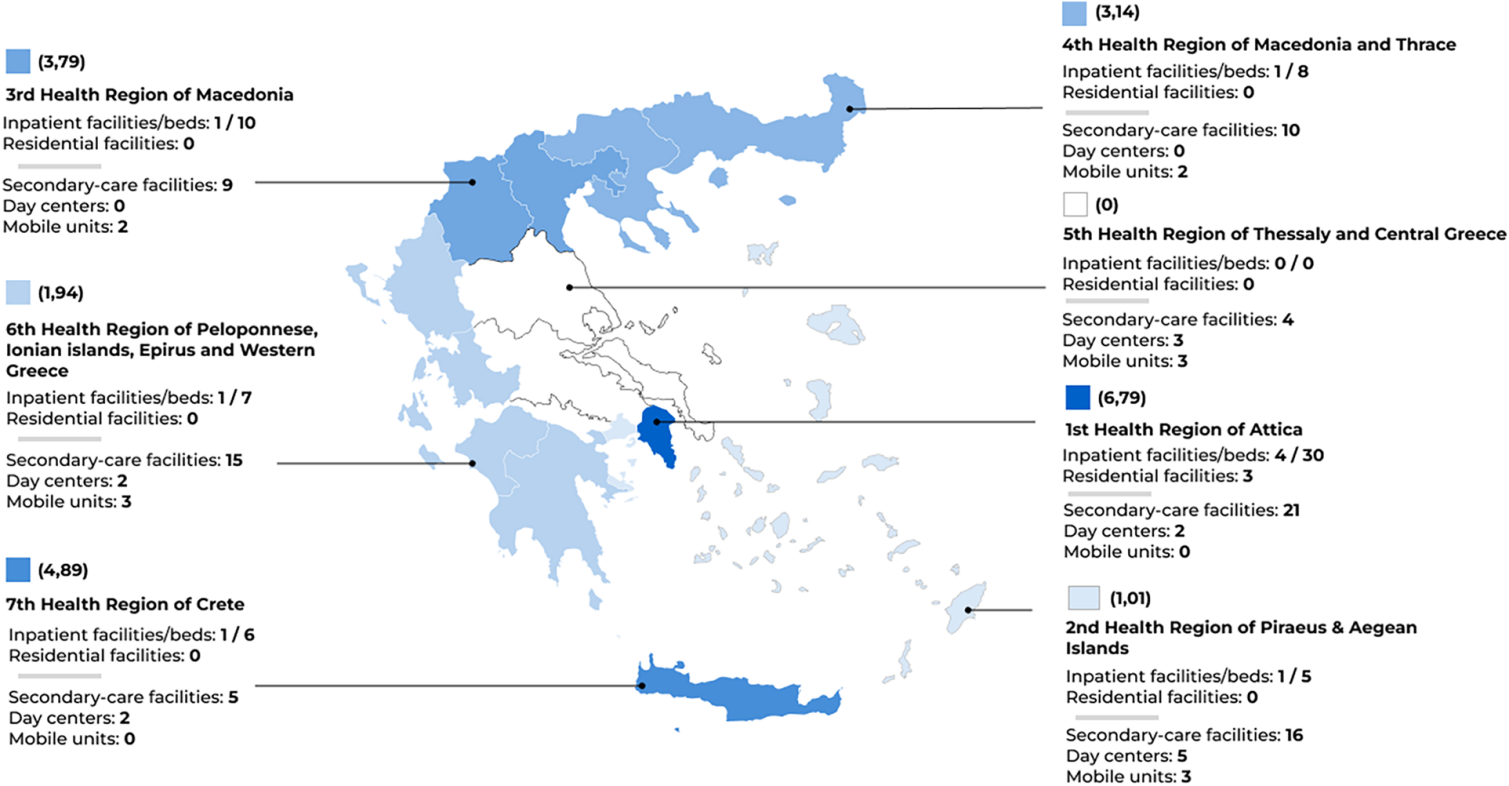
Mental health services for children and adolescents: regional distribution and density map of inpatient beds. Note: Color intensity represents the rate of inpatient beds per 100,000 people below 18 years (from 0 in Crete to 6,79 in Attica) converted to a 0 to 100 scale of saturation to the same color code.

The Hellenic Republic is a unitary state divided in thirteen regions and seven decentralized administrations. Although not exactly matching the administrative division in terms of geographical area, there are also seven regional health authorities (YPEs) for governing health care (see Figure 2) (39). Governance operates at three levels with shared responsibilities: the central government (Ministry of Health), the regional health authorities (YPEs), and municipal authorities (10). In practice, central governance concentrates the majority of attributions (40). While recent legislation transferred the organization of the primary care networks to regional and municipal authorities, their role remained limited, with most facilities still run by the Ministry of Health (41).

The ESY is defined as a primary care-oriented system. Nevertheless, to date, it does not operate in a community-oriented network of services, missing key elements such a dimensional, stepped-care approach, coordination of services, and focus on primary care and promotion (22, 41). Irrespective of location, patients can directly access health units aimed at primary and specialized care (10). Assistance is centered around specialists and hospitals, and there is no coordination across services or an unified register system (41, 42). There are only 1,200 general practitioners in the country, while a workforce of 5,000 professionals would be necessary to meet populational demands (41). Consequent to a lack of articulation and underfunding of public services, there are lengthy waiting lists for specialist consultations, resulting in an increased burden of out-of-pocket expenses for private alternative services. This process has been described as a passive privatization of the health sector (10, 43).

Over the past decade, legislation established a framework for primary care, yet there were barriers to its implementation (26, 44). The 2014 Law 4238 created the National Primary Healthcare Network (PEDY), a sectorized system that integrated clinics from Social Health Insurance, health centers, and contracted physicians (45). Following European Union recommendations, the 2017 Law 4486 further established two primary care structures: as a first tier, the Local Health Units (Topikes Monades Ygeias, TOMYs) would be located in neighborhoods to be the initial point of contact with the system, acting as gatekeeper and coordinator of care (41, 46). The next tier would be composed of Health Centers and PEDY units, managing conditions of increased complexity and also referring cases to specialized or tertiary services. However, there was an insufficient number of facilities and staff to widespread coverage, with reports of waiting times of up to three months before a primary care appointment (3).

In 2024, the Ministry of Health introduced a new plan to strengthen primary health care (47). It involves hiring 1375 primary care professionals and realigning the role of general practitioners to focus on promotion and prevention, management of chronic diseases, and improving health literacy. Additionally, the plan includes establishing seven to eight University Health Centers attached to Medical Faculties. These centers will be staffed by multiple primary care specialists, including two internal medicine/general medicine physicians (one specializing in emergency and the other in preventing cardiovascular diseases), one pediatrician (specializing in diabetes mellitus and obesity), and one psychologist with emphasis in Health Psychology. The centers will also offer telemedicine to outreach distant regions.

## 4 Mental health system

### 4.1 Historical development

In line with the global context, Greece has undertaken reforms aimed at replacing asylum institutions with a community-oriented mental health system (see Table 2 for legislative milestones) (48, 49). Changes can be traced back to the early 1980s, when the provision relied on secluded psychiatric hospitals. This was also the period of democratization in the country, with reflections on healthcare paradigms towards inclusive access and biopsychosocial approaches. In 1983, the National Health System Law 1397 set the stage for universal coverage and public provision of health services, also establishing the first Mental Health Centers (50). A starting point for the reform came about due to concerns at the Leros Mental Hospital, an overcrowded asylum-hospital in the island of Leros (51). The appalling conditions and severe violations prompted action from healthcare professionals and public authorities, leading to the 1984 European Community Regulation 815, which provided financial and technical support for the transition from asylum-hospitals to community-oriented services in Greece (52). Later on, the 1992 Law 2071 on the “Modernization and Organization of the Health System” further protected humaning rights with provisions for voluntary and involuntary hospitalization (53).

**Table 2:** Legislative and programmatic milestones of the Greek mental health reform.

Law 1397/1983	Foundation of the National Health System and the first Mental Health Centers
EEC Regulation 815/1984	The Commission of European Communities provided financial and technical assistance to Greece for deinstitutionalization and for improvement of conditions for mental health hospitalization
Law 2071/1992	Organization of the National Health System Provisions for voluntary and involuntary admission
Psychargos I (1997-2002)	The National Mental Health Plan was launched aimed at deinstitutionalizing mental health services and establishing a community-oriented model
Law 2646/1998	Classification of healthcare services into levels of care: primary, secondary, and tertiary
Law 2716/1999	Legal framework for the mental health reform, determining the organization of services based on the principles of sectoralization and community care, prioritizing primary and outpatient care, deinstitutionalization, psychosocial rehabilitation, and social reintegration
Psychargos II (2002-2007)	Second phase of the National Mental Health Plan
Psychargos III (2011–2021)	A further ten-year plan addressing ongoing challenges for establishing community-oriented services
Law 4461/2017	Enacting resolutions for the coordination of services, with administrative councils and committees at regional and sectoral levels Establishment of mental health sectors Enhancement of community participation in mental health policies
Joint Ministerial Decision *Γ*3*α*/*Γ*.*Π*.*οικ*.44342/2019	Organization and operation of Community Child and Adolescent Mental Health Centers, including provisions for universal access to psychosocial care at public structures and for the coordination and continuity of care
Mental health deputy minister	The Ministry of Health appointed a deputy minister for advancing mental health care
National Action Plan For Mental Health (2021–2030)	Defines an agenda for strengthening the community-oriented system in regards to sectorization and integration into primary health care, developing prevention programs, and advancing the structure for child and adolescent mental health services with a special focus on developmental conditions

In 1997, Greece unveiled Psychargos, the National Mental Health Plan aimed at deinstitutionalizing mental health services and transitioning towards community-based care (54). Focusing on human rights, mental health promotion, prevention, and rehabilitation, the 10-year plan was divided in phase I and II, each covering half a decade and achieving significant transformation. In 1999, the Law 2716 enacted the legal framework for the reform, legislating a community-based organization for services, the strengthening of primary and ambulatory care, and the closure of psychiatric hospitals (55). Importantly, it also introduced specialized mental health services to the elderly and to children/adolescents. Following this resolution, many asylums were substituted by an emerging infrastructure of mental health centers, day care units, child guidance services, psychiatric beds in general hospitals, open sheltered apartments, and rehabilitation units (48); a remarkable achievement was the closure of the asylum-like institution “Children”s Psychiatric Hospital of Attica”, which was in operation since 1960, transferring its departments to four general and pediatric hospitals (56). By 2002, sectorization had been successfully implemented, encompassing seven health regions and 37 mental health sectors (54).

In spite of achievements, barriers persist for fully accomplishing the objectives of the reform, including a lack of coordination across levels of care and a shortage of services and professionals, particularly in remote areas (see Figure 2, Table 3 and Table 4) (3, 62, 63). Over the last decades, successive initiatives have been established for solidifying the mental health care system and provision. In 2011, the Psychargos III was launched as a ten-year plan to strengthen the structure of community-based services, especially in undersupplied areas, organizing and monitoring the provision of services, supervising professional training and activities, and establishing promotion and prevention programs (64). In 2017, Law 4461 established scientific and administrative committees, councils, and coordination bodies at regional and sectoral levels, also enacting resolutions for enhancing community participation in mental health policies (65). In 2019, Law 2289 defined the organization of Community Child and Adolescent Mental Health Centers, providing universal access to comprehensive psychosocial care at public structures, also referring to the importance of coordination and continuity in care (66).

**Table 3:** Mental health workforce in Greece: physicians per speciality and region.

	Total population	Population under 19 years-old	Total number of physicians (rate per 100,000)^a^	Pediatricians (rate per 100.000)^b^	Child and adolescent psychiatrists (rate per 100.000)^b^	General Medicine (rate per 100.000)^a^	Psychiatrists (rate per 100.000)^a^	Neurologists- Psychiatrists^c^ (rate per 100.000)^a^
Total	10.482.487	1.946.707	68,469 (653,18)	3,857 (198,13)	406 (20,86)	3,524 (33,62)	1,701 (16,23)	150 (1,43)
Regional distribution								
Attica	3.814.064	687.248	31,415 (823,66)	1,614 (234,85)	242 (35,21)	784 (20,56)	892 (23,39)	82 (2,15)
Central Greece	508.25	90.019	1,806 (3,553,37)	118 (131,08)	3 (3,33)	201 (395,47)	25 (49,19)	5 (9,84)
Western Greece	648.220	123.971	3,297 (508,62)	215 (173,43)	19 (15,33)	270 (41,65)	51 (7,87)	1 (0,15)
Peloponnese	539.535	93.676	2,194 (406,65)	137 (146,25)	12 (12,81)	256 (47,45)	44 (8,16)	2 (0,37)
Thessaly	688.255	128.810	4,046 (587,86)	216 (167,69)	16 (12,42)	253 (36,76)	87 (12,64)	8 (1,16)
Epirus	319.991	56.440	2,454 (766,9)	149 (264)	6 (10,63)	159 (49,69)	55 (17,19)	1 (0,31)
Eastern Macedonia and Thrace	562.201	107.947	3,168 (563,5)	166 (153,78)	13 (12,04)	250 (44,47)	56 (9,96)	7 (1,25)
Central Macedonia	1.795.669	337.954	12,216 (680,3)	714 (211,27)	68 (20,12)	696 (38,76)	342 (19,05)	35 (1,95)
Western Macedonia	254.595	45.064	793 (311,48)	59 (130,92)	2 (4,44)	70 (27,49)	13 (5,11)	1 (0,39)
Ionian Islands	204.532	37.962	1,082 (529,01)	57 (150,15)	5 (13,17)	75 (36,67)	21 (10,27)	1 (0,49)
North Aegean	194.943	36.825	785 (402,68)	50 (135,78)	1 (2,72)	72 (36,93)	14 (7,18)	0 (0)
South Aegean	327.820	68.330	1,272 (388,02)	90 (131,71)	3 (4,39)	100 (30,5)	19 (5,8)	0 (0)
Crete	624.408	132.461	3,941 (631,16)	272 (205,34)	16 (12,08)	338 (54,13)	82 (13,13)	7 (1,12)

The workforce ratio was sourced from the 2,022 ELSTAT Health Professionals Survey (57). For population sizes, we consulted the 2021 ELSTAT population and housing census (58).

^a^ We calculated rations using the total population.

^b^ For child and adolescent psychiatrists and pediatricians, we calculated ratios using the under-19 population in Greece.

^c^ Neurology and psychiatry was a single speciality until 1981 (59).

**Table 4:** Distribution of child and adolescent mental health services in Greece.

Health region Child and adolescent mental health sectors (CAMH)	Estimated population under 18 years-old^a^	Inpatient units	Inpatient beds	Rate of inpatient bed per hundred thousand people under 18 years-old	Outpatient facilities^b^	Day centers^c^	Mobile Units	Residential rehabilitation facilities
Greece	2,190,643*	9	66	3,01	79	12	15	3
1st Health Region of Attica	441,973	4	30	6,79	21	2	0	3
2nd CΑΜΗ Sector of Attica	289,514	1	15	5,18	7	0	0	3
3rd CΑΜΗ Sector of Attica	152,459	1	15	9,94	14	2	0	0
2nd Health Region of Piraeus & Aegean Islands	492,971	1	5	1,01	15	5	3	0
1st CΑΜΗ Sector of Attica	197,963	1	5	2,53	8	3	0	0
4th CΑΜΗ Sector of Attica	199,224	0	0	0	5	2	0	0
CΑΜΗ Sector of Dodecanese	38,988	0	0	0	0	0	0	0
CΑΜΗ Sector of Cyclades	22,379	0	0	0	1	0	2	0
CΑΜΗ Sector of Lesvos - Chios - Samos - Limnos	34,417	0	0	0	1	0	1	0
3rd Health Region of Macedonia	264,192	1	10	3,79	9	0	2	0
1st CΑΜΗ Sector of the 3rd Health Region	207,403	1	10	4,82	8	0	1	0
2nd CΑΜΗ Sector of the 3rd Health Region	56,789	0	0	0	1	0	1	0
4th Health Region of Macedonia and Thrace	254,532	1	8	3,14	10	0	2	0
1st CΑΜΗ Sector of the 4th Health Region	154,307	1	8	5,18	5	0	0	0
2nd CΑΜΗ Sector of the 4th Health Region	100,225	0	0	0	5	0	2	0
5th Health Region of Thessaly and Central Greece “Sterea Ellada”	254,054	0	0	0	4	3	3	0
1st CΑΜΗ Sector of the 5th Health Region	143,864	0	0	0	3	2	2	0
2nd CΑΜΗ Sector of the 5th Health Region	110,190	0	0	0	1	1	1	0
6th Health Region of Peloponnese, Ionian islands, Epirus and Western Greece	360,185	1	7	1,94	15	2	3	0
1st CΑΜΗ Sector of the 6th Health Region	86,627	0	0	0	4	1	1	0
2nd CΑΜΗ Sector of the 6th Health Region	180,438	1	7	3,88	7	1	1	0
3rd CΑΜΗ Sector of the 6th Health Region	93,120	0	0	0	4	0	1	0
7th Health Region of Crete	122,736	1	6	4,89	5	0	2	0
CΑΜΗ Sector of the 7th Health Region	122,736	1	6	4,89	5	0	2	0

Some services may be limited in operation due to absence of specialized staff such as child and adolescent psychiatrists.

^a^ Official data regarding the population under 18 years-old at each health region is not available, as they do not match the administrative division used in the census. Our estimation was based on data from the 2018 Ministry of Health sectoral planning report, which draws on the 2011 census (60, 61). Differences are expected from actual numbers, as the sum of estimated population per region exceeds the national population size according to the census.

^b^ Outpatient services: include community centers, mental health centers, outpatient services of hospitals and universities, centers for outpatient treatment of specific conditions such as autism, and specific centers for speech therapy or learning disorders.

^c^ Day centers are day stays for people with autism or other conditions leading to functional impairment.

In 2020, the Ministry of Health appointed a deputy minister with exclusive responsibility for mental health, defining a comprehensive agenda for advancement (3). In February 2023, a new National Action Plan for Mental Health was released (67). Priorities include closing remaining long-term facilities and reforming forensic services and further expanding the community-based network, especially in regards to sectorization and integration with primary care; a goal was also set for reducing involuntary admissions to European Union standards. For promotion/prevention, it includes programs for workers’ mental health, anti-stigma campaigns, and integration of people with mental health disorders into the labor market. For special populations, the agenda involves a network of mental health services for children and adolescents, particularly the ones facing developmental conditions. A new legislation is also planned to reform the administration of mental health services, but it has not yet been finalized to be voted on in the Greek Parliament (68, 69). As revealed, a National Network of the Mental Health Services (*Ε*.*Δ*.*Υ*.*Ψ*.*Υ*.) will include all public and private mental health services, being divided into Regional Networks under the administration of the Deputy Manager of each YPE. The plan will establish a new National Organization for the Prevention and Management of Addictions including all related services operating across the country.

An overview of the current state of the mental healthcare system is provided by a 2021 joint assessment by the Deputy Ministry and the World Health Organization (WHO) (3). The report examined governance, organizational structure, service delivery, financial resources, and the workforce. Long-known issues were underscored, particularly the low availability and inequitable distribution of services and human resources, insufficient funding, and lack of coordination across different points of the system. Additionally, the report highlighted the absence of effective methods for monitoring performance, which were suggested as pivotal to establish funding mechanisms and directing payments. Tackling the need for distributing services according to community needs, the report recommended an assertive position from the Ministry of Health, as the primary funder of both public and private services, on determining where services should operate. It also highlighted the need for developing clinical protocols and care pathways tailored to individuals with mild, moderate, and severe mental health conditions, as well as prevention and promotion programs.

### 4.2 Organization and structure

The Greek mental health system is integrated into the broader healthcare system, with public health facilities managed by ESY, university hospitals hosted in ESY (except two in Athens under the jurisdiction of the Ministry of Education), private providers contracted by ESY, and private services directly contracted by users; all such facilities are subject to regulation by the Ministry of Health (3, 10). Additionally, the school system provides psychoeducational services focused on learning disorders (see next section). There are also mental health facilities administered by the Ministry of Defence, and forensic services operated by the Ministry of Justice, although they are not dedicated to children/adolescents. Philanthropic and non-governmental organizations (NGOs) and also participate in the mental health system, primarily operating residential-type facilities. For regionalization, a specific framework was created for mental health, with municipalities covered by Mental Health Sectors for adults (*Τ*.*Ο*.*Ψ*.*Υ*.) and for children/adolescents (*Τ*.*Ο*.*Ψ*.*Υ*.*Π*.*Ε*.) which operate under the seven YPEs through the Regional Administration of Mental Health Sectors (*Πε*.*Δι*.*Τ*.*Ο*.*Ψ*.*Υ*.) (39). In 2019, a ministerial decision established the current division of 17 child and adolescent mental health sectors (56, 65, 70).

Within this system, there are services spanning different levels of care, including special units of community services and residential assistance (see Figure 1) (3). Child and adolescent mental health services are integrated into this structure and can be found in inpatient and outpatient departments within the ESY and universities, community services, and also provided by NGOs and private mental health professionals. There are specialized units for children and adolescents or for autism/learning disorders, as well as general facilities that cater to children and adolescents (for instance, a few Mental Health Centers for Adults may include a child psychiatrist) (see Figure 1 and Table 3). Within primary care, pediatricians should be the main provider for this population, yet pediatric assistance is frequently sought in the private system and usually does not involve mental health assistance (41).

Some particular components of community mental health services are the Community Centers for Mental Health of Children and Adolescents (KOKEPSYPEs), the Hellenic Centers for Mental Health and Research (EKEPSYE), and the Mobile Mental Health Units (MMHU). The KOKEPSYPEs, formerly known as IPDs or Medical-Pedagogical Centers, are vinculated to existing mental health facilities in the ESY and address mental health and neurodevelopmental disorders (66). These units offer comprehensive psychiatric and psychological assessment, medication treatment, psychotherapy, psychosocial support, speech and occupational therapies, and special education services, being also responsible for community outreach as delivering psychoeducation/awareness programs in coordination with school institutions. For a catchment area of 250.000 population, a KOKEPSYPE should be minimally formed by four child/adolescent psychiatrists, three psychologists, three social workers, two occupation therapists, two health visitors (or community health agents), two speech therapists, one special educators, and administrative and general duties staff. For its turn, the EKEPSYEs are private organizations with several outpatient and rehabilitation units across the country, providing community services for small fees (approximately 2.50 euros for an appointment, and free-of-charge in cases of severe financial vulnerability) (71, 72). EKEPSYE units are supervised and funded by the Ministry of Health, and a plan was released in 2019 for increasing EKEPSYEs provision of child and adolescent mental health in rural areas of Greece, yet to date this was not implemented.

The MMHUs are designed to offer community-oriented care in rural/underserved areas, including early diagnosis and intervention, home care and crisis management, rehabilitation, family support, and anti-stigma programs (73, 74). They can be operated by public and university hospitals and non-profit NGOs, being composed of multidisciplinary teams with at least a psychiatrist, a psychologist, three nurses and/or health visitors, and administrative staff. In case of providing care to under 18 years-old, the team should include a child and adolescent psychiatrist. Services may be delivered either at patients' homes (with a MMHU car without logos of the organization) or at facilities provided by local authorities (such as health centers, rural clinics, schools, and other public buildings). For using existing infrastructure, MMHUs are considered low-cost services, and assessments indicate their effectiveness on reducing acute admissions (75). However, they face challenges related to understaffing and low-retention of personnel, particularly for MMHUs operated by public hospitals.

Existing upon the ESY framework, the mental health structure is under similar constraints in availability, regionalization, and coordination, with system inefficiencies resulting in prolonged wait times and pushing individuals towards out-of-pocket care (3, 10, 76). Mental health services were instituted based on the availability of private providers rather than community needs, then clustering in some specific urban areas and leaving peripheral areas unassisted; many YPEs do not count on inpatient units, so children need to be hospitalized in facilities far away from their homes (56, 63). While primary care should address mild to moderate mental health problems, in practice primary care providers are not involved in mental health care, lacking incentives and training for addressing this need (3). There is no gatekeeping mechanism and users tend to directly seek specialized providers. Services usually operate in isolation, as coordination mechanisms and patient pathways are lacking and an integrated electronic system is not available, meaning professionals cannot access registers from other facilities. On promotion and prevention, there is no cohesive mental health strategy, though some organizations and universities tap into ESY funding for anti-stigma/psychoeducation campaigns and the Ministry of Education promotes school-based campaigns addressing bullying and inclusivity (3, 63).

### 4.3 Available treatments for child and adolescent mental health

Services are provided in the public sector and at large at private providers with EOPYY reimbursement, including specialist consultation, medication, psychotherapy (individual, group, family), special educators, and speech, occupational, and physical therapy (3). Although public services are generally cost-free, low availability makes patients seek private providers, making reimbursement schemes the most frequent arrangement for care (see Table 5 for an overview of user costs). A standard fixed reimbursement amount applies to each service, which is not adjusted according to regional cost differences and is significantly below market prices (currently, there is no child and adolescent psychiatrist with an active contract with EOPYY for consultation, so reimbursement does not apply to this service). During times, repayment experience delays, as occurred during the Covid-19 pandemic.

**Table 5:** Reimbursement rates and average market price for mental health services.

Service	Average market price*	Reimbursement rate for private sector	User fee for afternoon outpatient visits in public services
Medical specialist consultation	€ 50–150	There is no active contract between EOPYY and child and adolescent psychiatry, making reimbursement unavailable for consultation	**University physicians** • Professor: € 72 • Associate Prof: € 60 • Assistant Professor: € 48 **ESY physicians** • Chief Director: € 64 • Director: € 60 • Consultant A: € 48 • Consultant B: € 36
Psychotherapy session	€ 30–70	15 € per session	Child and adolescent psychiatrists may provide psychotherapy within afternoon outpatient visits with the same rates as above
Special educator session	€ 20- 30	1.23 € per session	
Speech therapy Physical therapy Occupational therapy	€ 20–30	15 € per session	
Medications		Cost-sharing arrangement with user part of 0 to 25% (medications for psychosis are free of costs)	

* Average private prices were determined based on a search by a local coauthor, the range accounting to market factors including region, professional experience, and consultation procedure.

Service reimbursement caps are set based on diagnosis, yet not all relevant ICD-10 diagnoses are included. For instance, there is a maximum monthly value of approximately €510 for Autism Spectrum Disorder, €310 for Attention Deficit Hyperactivity Disorder, €120 for Emotional Disorders, €60 for Speech Sound Disorder, €210 for Childhood-Onset Fluency Disorder/Stuttering (being €90 euros for psychotherapy and €120 for speech therapy). Only two diagnoses are allowed, yet not summing reimbursement rates for overlapping therapeutics. Currently, the reimbursement limit is finally decided by EOPYY auditors on a case-by-case basis upon evaluation of the clinical presentation noted by the prescribing physician (e.g.,: auditors may reduce the number of reimbursed therapy below the maximum number set for the diagnosis). There are prerequisites for reimbursement, as a diagnosis/prescription by medical specialists providing child and adolescent treatments, such as child and adolescent psychiatrists, adult psychiatrists, neurologists (specialized in children), pediatricians (specialized in developmental and behavioral pediatrics), and, for some therapeutics, ophthalmologists and orthopedists. There is an appendix in the legislation specifying which specialty can prescribe for each diagnosis and the amount of each session.

### 4.4 Human resources and professional training

There are several professionals involved in child and adolescent mental health in Greece, including physicians, nurses, psychologists, social workers, speech therapists, occupational therapists, and health visitors. Table 3 provides an overview of the physician workforce as per the latest official survey (57). There are 406 registered child and adolescent psychiatrists in the country, corresponding to 20,86 specialists per 100,000 individuals below 19 (58). While the number stands above European Union averages, the actual ratio is shorter for significant parcels of the population, as the majority of professionals only work in the private system (3, 77). Furthermore, about 75% of all child and adolescent psychiatrists are concentrated in Attica (242) and Central Macedonia (74), with very few professionals in many regions (for instance, there is one specialist in North Aegean and two in Western Macedonia, amounting to a respective rate of 2,72 and 4,44 per 100,000 people under 19) (57). Other professional categories are not registered under a single association, and official data does not account for the total number of psychologists, nurses, social workers, occupation/speech therapists, and health visitors. Nevertheless, it has been reported that imbalances in distribution and understaffing extend to nursery personnel (78). While general hospitals tend to count on better staffing levels, mental health inpatient facilities face challenges in covering afternoon and night shifts, and community centers are compelled to prioritize essential tasks due to shortage of professionals (3).

In the realm of professional training and certification, child and adolescent psychiatry emerged as a separate medical specialty in 1981 and was previously categorized as a subspecialty of psychiatry (79). To align with the European Union of Medical Specialists, the residency program underwent changes in 2018 (80, 81). The present curriculum spans five years, with 3.5 years focused on field practice of child/adolescent mental health, including psychotherapy skills. However, the availability of training centers and positions for residents is limited, resulting in a five-year graduation rate of approximately 40 child/adolescent psychiatrists (3). General adult psychiatry training also extends for five years, including an optional placement of three months in child and adolescent services (79, 82). In the absence of child and adolescent psychiatrists, general adult psychiatrists should provide services to this population regardless of limited training. In practice, this is infrequent except for older adolescents and mainly in the private sector. However, due to the very limited number of inpatient beds especially for adolescents aged 14–17 years, a considerable number of older adolescents are hospitalized in adult psychiatric departments.

In academia, the field of child and adolescent psychiatry is still under consolidation (56). Out of seven medical schools in Greece, only four have faculty members specializing in this area. Of those, only one integrates a child psychiatry discipline in the core curriculum, whilst the other three offer an optional course. As a result, a significant number of graduate physicians lack fundamental knowledge to address children's mental health issues. Moreover, pediatricians' residency curriculum should include mental health theoretical courses, yet placements in clinical settings are scarce (56, 83).

As for psychologists, a university degree is required to obtain a practice permit after a four year undergraduate training (84). There are options for post-graduate specializations, such as clinical psychology, school psychology, or applied cognitive and developmental psychology. However, there is no formal requirement for specialist practice with children and adolescents. Vacancies in child/adolescent services refer to psychologists in general, therefore any psychologist can apply regardless of specialization or experience with minors. As for nursing, a previous legislation established the specialization of mental health nurses, which has been recently expanded and covers theoretical and practical training in ESY services (3, 85, 86). Nurses in Greece undergo four years of university education, with another two years dedicated to their chosen specialty. With the new program in place, nurses are now able to directly pursue mental health nursing as their specialization. Notably, in inpatient facilities, about 90% of nurses are specialized in mental health. Furthermore, there are ongoing programs for continued education, with approximately 500 nurses having received training in community care. Other training programs encompass areas like forensic care, drug addiction, and internet addiction.

### 4.5 Clinical practices and quality of care

The standards of care differ considerably across both public and private facilities in the country, and provision is not regularly monitored using indicators of quality (48). There is a gap in standardized clinical protocols defining practices for mental health conditions according to severity, as existing guidelines are often narrowed in scope and not widely disseminated (3). Professional practices are inconsistent, as treatment approaches are typically determined by individual professionals and usually influenced by tradition or personal preference rather than evidence-based protocols. Additionally, interviews with professionals from diverse backgrounds exposed gaps in skills such as psychotherapeutic techniques, case management, aggression management and violence de-escalation, and human rights safeguarding (3). While gaps were identified, the absence of a systematic monitoring of services means that the actual standards of care remain largely unknown (56). The funding model compounds this issue: resources are distributed according to the reported provision of services or block grants, without allocation mechanisms based on quality indicators (10).

Field assessments have suggested substantial deficiencies on interdisciplinary teamwork and community-based approaches, with many professionals adhering to a medical-dominated model in the central figure of a psychiatrist (3, 63, 87). There are fragilities in the community-based services, where timely interventions are hindered by long waiting lists, unavailability of care in certain areas, and inadequate coverage (3). In certain less-populated regions, such as Thrace and Ioannina, reports suggest that services are more effectively coordinated within a sectorized and community-oriented framework, which is evidenced by decreased rates of involuntary admission among the adult population (contrasting to alarmingly high rates across the country) (3, 88, 89). Moreover, even in regions with limited mental health units, there is a lack of formal connection between child/adolescent services and adult mental health services. Therefore, transition of adolescents to adult care usually is self or family driven, or by personal communication between mental health professionals rather than protocols and guidelines. This further leads to fragmentation in care, particularly for young adults suffering from Autism Spectrum Disorders.

### 4.6 Mental health at the educational system

Some mental health services are provided at the educational system, primarily focusing on learning disorders and offered through the Centers for Interdisciplinary Assessment, Counseling, and Support (KEDASY) (90, 91). In Greece, education across all levels is tuition-free, financed by the Ministry of Education, Religious Affairs and Sport (92). The system operates in a regionalized, decentralized structure with three levels of administration: local municipalities, thirteen regional directorates, and central government (Ministry of Education) (93). Every Regional Directorate of Education is staffed with an Advisor of Education, who is responsible for providing pedagogical and scientific guidance for educators, special educators, psychologists, social workers, and other specialties working in schools and KEDASYs (91). KEDASY centers are responsible for promoting awareness of psychosocial issues, assisting the implementation of mental health promotion and prevention, and offering psychoeducational support for children and adolescents with special educational needs and/or disabilities (91, 92). At a minimum, each center comprises a special education teacher, a psychologist, a social worker, and occasionally, a speech, occupational, or physical therapists.

The School Networks of Educational Support (SDEYs) is the network of schools built to promote access to education, cooperation across services, and protection and promotion of psychosocial health of students (94). Each SDEY is built at a local level and serves up to five general/special schools, being connected to one regional KEDASY as the next-tier supporting service. At each school unit, a School Life Advisor collaborates with the Advisor of Education and KEDASYs to provide guidance to students and caregivers on psycho-pedagogical issues, such as prevention and management of extreme behaviors, learning difficulties, and inclusion and integration (95). Additionally, a Committee of Interdisciplinary Support (EDY) is instituted for looking after the psychosocial wellbeing of students, composed of members of the school (directors and special support teachers) and from the affiliated SDEY (psychologists or social workers) (94, 96). The EDY is in systematic articulation with KEDASY in regular and extraordinary meetings, also collaborating with other health, educational, and legal-judicial services, the latter especially in instances of child maltreatment and neglect. Within this structure, the psychologist evaluates needs, offers counseling to students and families, and implements individual or group interventions targeting inclusion, awareness, and stress mitigation (94). For its turn, the social worker addresses socio-contextual challenges that may influence students and their families.

There are delineate pathways for students with special needs and psychosocial challenges (96). Initially, the EDY is responsible for identifying such students, addressing concerns within the school resources. If issues persist, the student is referred (either by the EDY, by schools without EDY, and by caregivers) to a KEDASY center for a comprehensive psychoeducational evaluation (alternatively, the student may be referred to a public mental health service for establishing a medical diagnosis) (91, 97). Based on those, students may be directed to special education services or supportive guidelines may be provided for care at school (91). Special services are categorized by group involvement and severity: Integration Class (TE) offers small-group interventions within general schools, catering to students with preserved functionality who face learning or intellectual difficulties. Parallel Support (PS) refers to co-teacher intensive one-to-one assistance within the general school, which is designed for children that are able to follow the regular class program with adequate support, when special education schools are unavailable, or to attend KEDASY recommendations. Lastly, Special Education Schools (SMEAE) are reserved for children with disabilities that impair their capacity to attend regular schools. Typically, these institutions provide specialized services for children diagnosed with Intellectual Developmental Disorder, Autism Spectrum Disorder, cerebral palsy, hearing impairments, and mobility disabilities.

This structure also faces operational challenges, including a pressing shortage of psychologists across schools, special schools, and KEDASYs (98). As a result, EDY committees often cannot serve all schools, leading to *a priori*tization of special education services. At regular schools, the scarcity of specialists leads to irregular visits, compromising psychosocial diagnostics and interventions (99). KEDASY evaluations are characterized by prolonged waiting times, often surpassing a school year. Due to the lack of physicians, it is common that KEDASYs refer children to ESY services for a diagnosis before their final report, enhancing delays as mental health facilities face prolonged waiting lists. Similarly, the demand for SMEAEs far exceeds the capacity of provision, and such units often lack technical capacity for delivering adequate specialized services.

Worthy noting, lesser-known structures related to wellbeing and mental health are the Centers for Creative Work for Children (KDAP) which operate under the new Ministry of Social Cohesion and Family (100). These facilities roll out programs aimed at fostering socialization, education, communication, and sensorimotor training, typically through recreational and cultural activities. KDAPs are staffed by educators, artists, physical therapists, and social workers, catering to both Greek and migrant children with typical development or with disabilities. For the latter, there are also specialized Centers for Creative Work for Children for Children with Disabilities (KDAPmeA).

## 5 Service mapping: availability and distribution

The infrastructure for mental health care is considered inadequate in terms of availability and distribution, leading to service clusters and neglected areas (3, 77, 101). The fragility is accentuated in regard to child and adolescent facilities, as only 30% of planned services have been implemented (56). As per a 28-country survey in Europe, Greece was ranked at the lower range of coverage of mental health services per hundred thousand young people (102). Indeed, the figures are scarce: according to the WHO Mental Health Atlas 2020, there were only 12 inpatient facilities specifically for children and adolescents in the country (103). A 2019 report further indicated only 34 inpatient beds for ages 0–15 years and 28 beds for ages 14–17 years, leading to cases of hospitalization in adult departments (56). Outpatient care was also deemed insufficient, locating only 26 primary care units assisting children and adolescents throughout the country (3). While overall statistics raise concerns, they may fluctuate based on methodologies for accounting for services; a more fine-grained account is provided by the Ministry of Health's report on the sectoral planning for the development of Mental Health Units, individually gathering data on all mental health facilities from September 2017 to June 2018 (60). Compared to international benchmarks, consistent gaps were found across the country: achieving adequate coverage would require an additional of 1200 professionals and 208 services, including 28 facilities for children and adolescents.

For an up-to-date situational analysis, we mapped public and philanthropic mental health and psychoeducational services for children and adolescents in each health sector between December, 2021 to May, 2022 with continuous revision throughout 2023 and 2024 (see Table 4, Table 6, Figure 2). We initially collected data at governmental and regional websites and then directly reached out to facilities for gathering information on service's type, location, operational status, and contact information (in the case of special schools, we have only consulted official lists). Next, experts from each health region revised the table and have been continuously checking updates. Full datasheets can be consulted at our open repository (https://osf.io/crz6h/) (104). Information on available services is also delivered to the community through a user-friendly online interface (https://camhi.gr/en/mapping-mental-health-and-psychoeducational-services-in-greece/) (105).

**Table 6:** Psychoeducational services for children and adolescents in Greece.

	KEDASY	Special school units
Greece	71	479
Regional Directorates of Primary & Secondary Education		
Eastern Macedonia and Thrace	5	30
Attica	13	99
North Aegean	4	16
Western Greece	4	38
Western Macedonia	4	19
Epirus	4	20
Thessaly	4	41
Ionian Islands	4	15
Central Macedonia	10	87
Crete	4	38
South Aegean	5	24
Peloponnese	5	25
Central Greece	5	27

Note: The regional division is under the governance of the Ministry of Education and aligns with the 12 regions of Greece, thus it does not correspond to the Child and Adolescent Mental Health Sectors established for healthcare. There is usually one Kedasy per prefecture, except in Attica where Athens prefecture has more than one.

To date, we collected data on 79 outpatient facilities, 12 day centers, 15 mobile units, 9 inpatient units (with 66 beds), 3 rehabilitation houses, 72 KEDASY centers, and 479 special schools. Worthy noting, a few outpatient facilities were not supplied with a child and adolescent psychiatrist, either due to temporary understaffing or absence of vacancies. Using service-level data, this analysis clearly delineates the disparities in provision across the country, particularly in inpatient units: only seven out of 17 child and adolescent mental health sectors count on inpatient beds, and almost half of inpatient facilities are concentrated in the 1st Health Region of Attica. The 5th Health Region is not supplied with any inpatient services, and populated regions such as 6th and 7th Health Regions rely only on 7 and 6 beds, respectively. Moreover, while the 3rd and 4th Health Regions register inpatient beds, all these facilities are actually located in the same town of Thessaloniki. To a lesser extent, outpatient care also encounters marked imbalances in distribution. The 1st Health Region of Attica concentrates 21 secondary-care facilities, yet its second sector is not supplied with day centers for autism as they are all located in the third sector. In contrast, the 5th Health Region has only four outpatient facilities to serve a population exceeding half of Attica's.

Notably, some figures in our service mapping differ from previous reports. For example, while we identified 9 inpatient units, the 2020 WHO Mental Health Atlas reported 12 facilities based on government data (103). This discrepancy is likely due to our use of a service-level outreach analysis in contrast to reliance only on administrative data. In several cases, facilities listed in official catalogs were either non-operational, had closed, or lacked the necessary professional staff to function.

## 6 Recommendations for improving strategic points

In this section, we review potential strategies for addressing caveats within the Greek child and adolescent mental health system. Noteworthy, many of the issues have been spotlighted and targeted before, including ongoing efforts as per the agenda set by the Mental Health Deputy Ministry (67). Yet, several implementation hurdles are inherent to complex systems influenced by economic, financial, social, and political dynamics. For instance, the joint WHO and Ministry of Health assessment emphasized the need for a more proactive role of central governance in determining the location of services, which is an intricate agenda involving multiple stakeholders and a political front (3). While this strategy may constitute a crucial point for advancement, we consider its intricacies beyond the reach of the present report. Instead, we adopted a technical standpoint as per Kousoulis et al. (106), reviewing international practices that could be tailored to the Greek context. Recognizing funding constraints, we aimed at recommendations enhancing system efficacy and optimizing resource allocation.

### 6.1 Strengthening mental health access in primary care

#### 6.1.1 Current scenario

The development of a solid primary care system is still underway in Greece. Both health professionals and the general public exhibit skepticism towards primary care, and while a framework has been set up for establishing services such as TOMYs and PEDYs, they remain limited in number and operation (26, 42, 107). This way, mental health conditions are usually not addressed at primary care, which is bypassed by individuals directly reaching specialists irrespective of severity criteria. The recent ministerial plan to enhance primary care may potentially advance provision, even though a pathway was not specified for child and adolescent mental health (47). To date, pediatricians more often work in private settings and are not involved in mental health assistance, lacking practical training or incentives to address this need (3, 41). As child and adolescent mental health is not consistently included in medical school curriculums, many physicians providing primary care assistance lack essential skills in this area (56).

#### 6.1.2 Rationale and strategies

Primary care pediatricians should identify mental health issues and streamline access to specialized care (108, 109). Despite resistance to primary care being a historical challenge worldwide, strategies such as medical training initiatives and financial incentives have helped to foster a culture and workforce of general practitioners and rural doctors in several countries (110–114). For pediatricians, rotations in psychiatry services knowingly improve the perceived responsibility and competence in catering to child and adolescent mental health (115–117); a core practice training in residency curricula could capacitate future pediatricians, and there are available frameworks for the development of a mental health curriculum with emphasis in multidisciplinary care (118–120).

As for current workforce challenges, Child Psychiatry Access Programs (CPAPs) have emerged as initiatives to support primary care providers in addressing child and adolescent mental health (121–123). CPAPs may have multiple components, including training programs, education resources and clinical protocols, and the promotion of collaborative and matrix-based care models. Capacity building through training have proved useful in enhancing quality outcomes in pediatric mental care, as well as improving the competences of professionals and the satisfaction of service users (124–128). For its turn, integrated care models are effective for improving assistance for several child mental health conditions, and may include specialist-primary care liaison with onsite or remote supervision, the latter particularly promising for underserved areas (123, 129, 130). In this direction, the use of digital technologies is encouraged to support training and supervision, and may also include telepsychiatry consultation where specialist referrals are unavailable (108). Currently, the CAMHI is implementing a nationwide training program for pediatricians, focusing on mental health assessment, communication skills, first-line interventions, and referrals (131). The program is based on context-sensitive and adaptable asynchronous modules, as well as supervision sessions with specialists. Currently in its pilot phase, the training program will be available for ongoing implementation and may support capacity building in the country. Additionally, a digital platform for nationwide supervision and technical support is under development, and could serve as a basis to scale up integrated care models.

### 6.2 Coordination of care

#### 6.2.1 Current state

The mental healthcare system faces significant challenges in coordination and continuity of care (3, 10, 63). The absence of a referral system or electronic record platform means professionals cannot access patient histories across different service points. Roles and location of providers are not defined within a unified strategy, and the fragmentation of care results in overlapping services and duplicated efforts. Coordination mechanisms are missing, including case management, team communication, discharge communication protocols, and joint treatment planning.

#### 6.2.2 Rationale and strategies

Shared care between primary and specialty level is currently considered a best practice model for managing conditions such as depression, with stepped care recently consolidating as the prime framework for mental health systems (108, 132). Based on escalated interventions according to identified needs, stepped care tends to optimize resources by defining patient pathways and the roles of the different levels and providers (132, 133). Typically, mild to moderate cases should be managed in primary care, with a resolution rate of over 90% for conditions such as depression and anxiety; referrals are reserved for more severe conditions or those unresponsive to initial treatments (19, 134).

There are a number of models to implement mental health stepped care, commonly aiming at early case identification, rapid access, and continued care (135–137). For children and adolescents, recommendations include tailored steps for age groups, special mechanisms for disadvantaged populations, and individualized identification of needs (133). Although patient pathways vary, primary health care as the main entry door usually stands as a key element for system efficacy (138, 139). There is no fit-for-all model, and the coordination of services varies based on local contexts (19, 140, 141); yet, effective communication mechanisms are common attributes to ensure continuity of care and transition across services (142). In this sense, it has been shown that an integrated electronic health record and referral system optimizes resources (112, 143); when implementing such a system, challenges to be considered include minimizing the additional workload for health professionals and avoiding fragmentation of software platforms across various types of providers and services (144–146).

### 6.3 Establishing standards of care and monitoring performance

#### 6.3.1 Current state

currently, professional practices are often determined by local traditions or individual choices, as standards of care were not established across different providers operating in the Greek mental health system (3). Without a systematic monitoring of performance, EOPYY is frequently unaware of the quality and efficiency of services they contract; funding is predominantly based on historical budgets or provision of services, without any performance-based adjustments to payment.

#### 6.3.2 Rationale and strategies

Establishing standards of care involves constructing clinical protocols to guide assessment and treatment of various conditions according to evidence-based, cost-effective practices; the adherence to such guidelines has been consistently associated with improved outcomes (147, 148). For mental health care, there are a number of useful sources for clinical guidelines (149, 150). Noteworthy, protocols should be adapted to be context-sensitive to reflect local culture and the availability of resources at each region, considering an inclusive agenda that recognizes needs of diverse communities (109, 151).

On the other hand, collecting indicators help to gauge the results of health systems, measuring their alignment with quality standards (152). There are some frameworks for designing performance monitoring for mental health assistance, including specialized child and adolescent care (152–154). With marked similarities, they are usually compounded by domains as safety, effectivity and efficacy, appropriateness, timely response, equitable access, continuity/integration, structure and human resources, and populational health outcomes. So as to align with the WHO concept of measuring responsiveness to population needs, recommendations include involving stakeholders such as health professionals and people with lived experience of care (152, 155, 156). A list of 42 potentially useful metrics for mental health is provided by a study that compared health monitoring systems across eight countries, deriving a set of widely-employed indicators considered both nationally-consistent and locally-relevant for services (157). In some cases, indicators are tied to financial incentives or set as prerequisites for funding allocation, with systematic reviews suggesting that pay-for-performance enhances mental health care outcomes (158, 159).

## 7 Strengths and limitations

This is a comprehensive review with a thorough examination of various sources, including academic articles, guidelines, policy statements, and legal documents, in addition to a mapping of available structure collecting service-level data. Our research team was composed of international and local expertise from different backgrounds to ensure multiple perspectives were considered when evaluating the health care system. However, narrative reviews present significant limitations; while valuable for providing an in-depth assessment of a specific topic, they are prone to bias on document selection and to the subjective viewpoint of authors. Concerning the service structure assessment, another limitation is the absence of data on staffing, precluding an analysis of indicators on available professionals per health sector. We opted to not collect this data for its inaccuracy, as we could not ascertain professional hours dedicated to services (e.g.,: full or partial-period) and there were variations of personnel over short periods of time.

## 8 Conclusion

This review synthesizes the child and adolescent mental healthcare within the Greek health system, also mapping available services per health region for a structure assessment. The mental health system is integrated into the National Healthcare Service (ESY), with some facilities connected to the educational system. Through continued efforts since the psychiatric reform, an initial framework for community-oriented mental health care has been established in the country, including regionalization of services and universal coverage. However, practical challenges persist for its full realization, including a limited availability and understaffing of services within the public system, significant underprovision in many regions, limited coordination across providers, and lack of standards of quality and monitoring mechanisms. Strengthening mental health care in primary health centers and implementing stepped-care approaches are highlighted as necessary strategies to improve access and optimize the system's efficiency.
